# New Method of Reducing Dislocations of the Shoulder

**Published:** 1871-05

**Authors:** 


New Method of Reducing Dislocations of the Shoulder. —Dr. Samuel Logan, of New Orleans, describes his method. The patient is placed on the back, while the surgeon, having removed his boots, seats himself so that his legs shall be nearly at right angles to the patient's body, but pointing rather upwards. He places the heel of one foot in the armpit, against the ribs, so as to press a little crosswise; the base of the great toe of the other foot is placed against the acromion process, and the surgeon pushes with moderate force while traction is made upon the arm.—*Transactions Am. Med. Association.*

